# A biomarker basing on radiomics for the prediction of overall survival in non–small cell lung cancer patients

**DOI:** 10.1186/s12931-018-0887-8

**Published:** 2018-10-10

**Authors:** Bo He, Wei Zhao, Jiang-Yuan Pi, Dan Han, Yuan-Ming Jiang, Zhen-Guang Zhang, Wei Zhao

**Affiliations:** 1 Department of Medical Imaging, the First Affiliated Hospital of Kunming Medical University, No.295 Xichang Road, Kunming, 650032 Yunnan China; 2 Department of Thoracic Surgery, the First Affiliated Hospital of Kunming Medical University, Kunming, 650032 Yunnan China; 3 Department of Pathology, Kunming Medical University, Kunming, 650500 Yunnan China

**Keywords:** Non-small cell lung cancer, Radiomics, CT, Random forest, Survival status

## Abstract

**Background:**

This study aimed at predicting the survival status on non-small cell lung cancer patients with the phenotypic radiomics features obtained from the CT images.

**Methods:**

A total of 186 patients’ CT images were used for feature extraction via Pyradiomics. The minority group was balanced via SMOTE method. The final dataset was randomized into training set (*n* = 223) and validation set (*n* = 75) with the ratio of 3:1. Multiple random forest models were trained applying hyperparameters grid search with 10-fold cross-validation using precision or recall as evaluation standard. Then a decision threshold was searched on the selected model. The final model was evaluated through ROC curve and prediction accuracy.

**Results:**

From those segmented images of 186 patients, 1218 features were obtained via feature extraction. The preferred model was selected with recall as evaluation standard and the optimal decision threshold was set 0.56. The model had a prediction accuracy of 89.33% and the AUC score was 0.9296.

**Conclusion:**

A hyperparameters tuning random forest classifier had greater performance in predicting the survival status of non-small cell lung cancer patients, which could be taken for an automated classifier promising to stratify patients.

**Electronic supplementary material:**

The online version of this article (10.1186/s12931-018-0887-8) contains supplementary material, which is available to authorized users.

## Background

Lung cancer, one of the highest risky cancers, is the leading cause of cancer death with a high mortality rate of 82.3% in 5 years after diagnosis (National Cancer Institute). [Noone AM, Howlader N, Krapcho M, et al. SEER Cancer Statistics Review, 1975–2015, https://seer.cancer.gov/csr/1975_2015/] Non-small cell lung cancer is a subtype lung cancer, which accounts for 85% among lung cancers [1]. The 5-year survival rate decreases dramatically as the cancer entering advanced, from 40% for stage I to only 1% for stage IV [2, 3]. It was reported that CT texture analysis could be helpful to further classification of treatment as it provided information on the intratumor heterogeneity [4] which might be the reason for disparate outcomes of patients. Thibaud P. Coroller and etc. used 7 radiomic features to predict pathological response after chemoradiation [5].

In the past 10 years, medical digital image analysis has grown dramatically as advancement of the pattern recognition tools and increase of the data collection. Radiomics offers unlimited imaging biomarkers which are promising to help cancer detection, prognosis and prediction of treatment response [6, 7]. With the high-throughput computing, it’s possible to quickly extract various quantitative features from digital images such as MRI and CT. Since cancers are more likely to be temporal and spatial heterogeneous, the use of biopsy might be limited. Furthermore, medical digital imaging could give a whole picture of the tumor shape, texture and volume, and it is also a noninvasive way to get comprehensive tumor information [8]. Some researches indicated that there was a relationship between radiomic features and tumor grades, histology, metabolism, and patient survival and clinical outcomes [9–11]. Kitty Huang et al. also found high risk CT features were significantly associated with local recurrence [12]. Chintan et al. studied the prognostic characteristics of radiomic features between lung cancer and head & neck cancer and found association among 11–13 features and prognosis, histology and stage [13]. Jiangdian et al. investigated the prognostic and predictive ability of phenotypic CT features in non-small cell lung cancer patients and reported an overall clinical stage prediction accuracy of 80.33% [14]. Those previous studies have shown that medical image analysis has a promising ability in improving cancer diagnosis, detection, prognostic prediction on oncology [8].

With those antecedent studies, radiomics displayed its hopeful and cost-effective potential in the area of precision oncology. Even though there have been already numerous researches on the prediction of cancer diagnosis or stage classification, most of them used default parameter or manual selection which might not efficient enough. Most of the time default parameters could give us great result, but the ability of the model would be maximized by parameters optimization when we conduct the training stage [15]. This study intended to construct an automatic grid search [16] hyper-parameters tuning classifier to make detections on the survival status of non-small cell lung cancer patients based on the radiomics features. The dataset was randomized into training set and validation set. A random forest classifier with hyperparameters tuning was used to make classification of survival status of non-small cell lung cancer patients in training set. The model was assessed on the validation data by ROC curve as well as the prediction accuracy.

## Methods

### Data sets

The study included 186 non-small cell lung cancer (NSCLC) patients from two merged datasets R01 and AMC. The patient characteristics and CT images were obtained from the cancer imaging archive (TCIA) database (10.7937/K9/TCIA.2017.7hs46erv). Clinical data of all 186 NSCLC patients are provided in Table 1, including the gender, smoking history, histology, treatment, and overall survival data. Additional file 1: Table S1 shows detailed staging information.

**Table 1:** Demographic characteristics

Characteristic	Number of Patients (%)
Gender	
Male	120 (64.5%)
Female	66 (35.5%)
Smoking Status	
Nonsmoker	39 (20.9%)
Former smoker	117 (62.9%)
Current smoker	30 (16.2%)
Histology	
Adenocarcinoma	154 (82.7%)
Squamous cell carcinoma	29 (15.6%)
NOS	3 (1.7%)
Treatment	
Surgery	33 (17.7%)
Chemotherapy	40 (21.5%)
Radiotherapy	19 (10.2%)
Adjuvant Treatment	40 (21.5%)
Unknown	54 (29.1%)
Overall Survival	
Dead	37 (19.9%)
Alive	149 (80.1%)

*NOS* not otherwise specified

This table displayed the clinical data of all 186 NSCLC patients, including the gender, smoking history, histology, treatment, and overall survival data

### Feature extraction

One thousand, two hundred eighteen tumor characteristics were quantified by extracted features from the lesion segmented from patients’ CT images. The radiomic features can be categorized into four types such as intensity, shape, texture and wavelet. An open-source package in Python, Pyradiomics was used for various features extraction from CT images [17]. A list of 50 quantitative features including first order features, shape features, Gray Level Size Zone Matrix (GLSZM) features, Features Gray Level Run Length Matrix (GLRLM) features and etc. were extracted. The shape descriptors were extracted from the label mask and also not associated with gray value.

### Data balance and data splitting

Extracted features were then weighted differently as a result of data balancing. In machine learning, algorithms assume the distributions of groups are similar. In practice, when the disproportion of classes happens, the learning algorithms tend to be biased towards the majority class. But in this study, we are more interested in the minority class with more adverse events take place [18]. Due to sample imbalance (the number of being alive is much less than that of being dead), rather than simply applying over-sampling with replacement for data balance, we conducted a synthetic minority over-sampling (SMOTE) method to increase the size of the minority group. SMOTE can be used when the number of the category is larger than 6 since it generated the new examples by taking samples of the feature space for the target class and its 5 nearest neighbors. SMOTE has an advantage of making the decision region of the minority class more general [19, 20]. The final size of the dataset was *n* = 298. Because the minority group was oversampled thus the dataset was randomized into training set (*n* = 223) and validation set (*n* = 75). The distribution of being alive and dead between training and validation set was also plotted.

### Classifier construction

Based on the radiomics features, we aimed to build a radiomics-based survival status prediction model using random forest classifier and hyperparameters tuning in Python [21]. Random forest creates multiple decision trees by randomly choosing subsets of features to make the classification based on the mode (for classification) or the mean (for regression) from all the smaller trees [22, 23]. It has the advantage of being less vulnerable to overfitting problem compared to decision-trees. A generic random forest classifier was constructed first. Parameter estimation using grid search with 10-fold cross-validation was applied to the training data for parameters tunings such as the number of features to consider for the best split (max_feature), the number of trees in the forest (n_estimators), the maximum depth of the tree (max_depth), the minimum number of samples required to split an internal node (min_sample_split). Precision and recall score were used as evaluation standard for parameters tuning respectively. The two best models with different optimal hyperparameters can be acquired by the top mean precision or recall score respectively. The final preferred model was selected by comparing their performance on the validation data.

### Decision threshold adjustment

Instead of directly adopting the absolute predictions, this study also applied for decision value tuning to balance the trade-off between precision and recall. A function of decision values was used to determine the decision threshold of the chosen model to maximize the precision with high recall.

### Radiomics model assessment

Model performance was evaluated in terms of the operator characteristic curve (ROC), the area under the curve (AUC) and accuracy, which could quantify the prediction performance of the classifier model.

## Results

### Class distribution

**Fig. 1:**
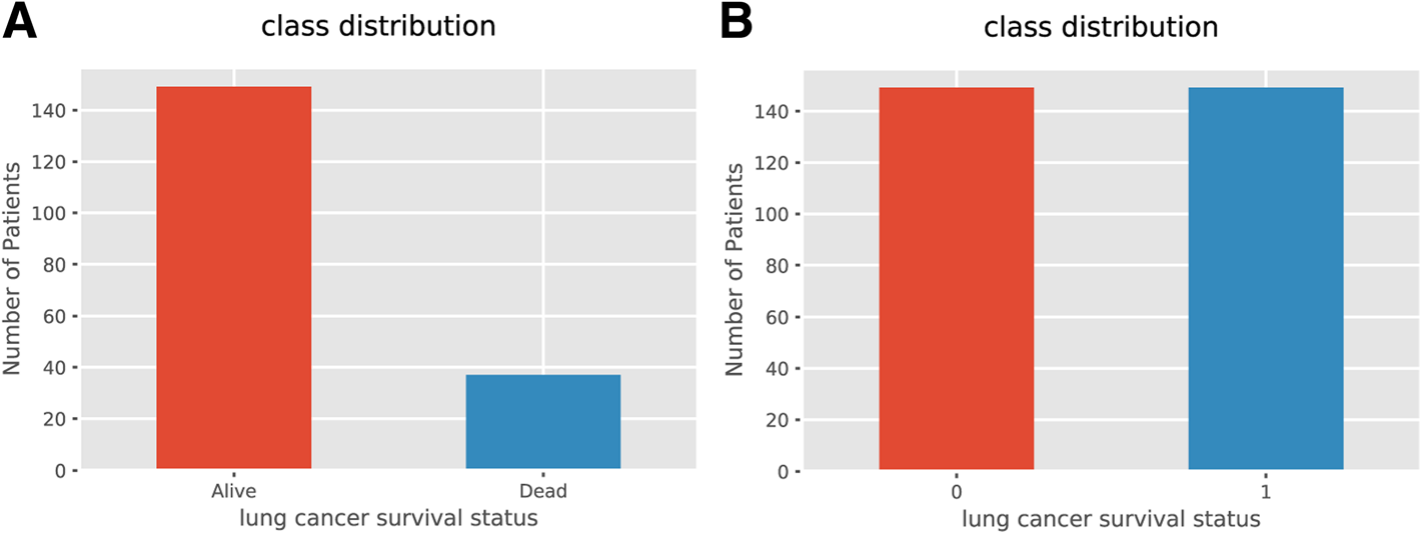
Survival Status Distribution of Patients. **a** Before oversampling. **b** After oversampling. X axis is the survival status of the patients: the blue bar represents the alive group and the orange bar represents dead group; Y axis is the number of patients in each survival group

The class distribution of each class before (Fig. 1a) and after oversampling (Fig. 1b) were presented. It was obvious that after SMOTE method, the number of patient being dead was similar to the number of those being alive. To be note, the sample size after oversampling was 298.

### Construction of the survival status prediction model

**Fig. 2:**
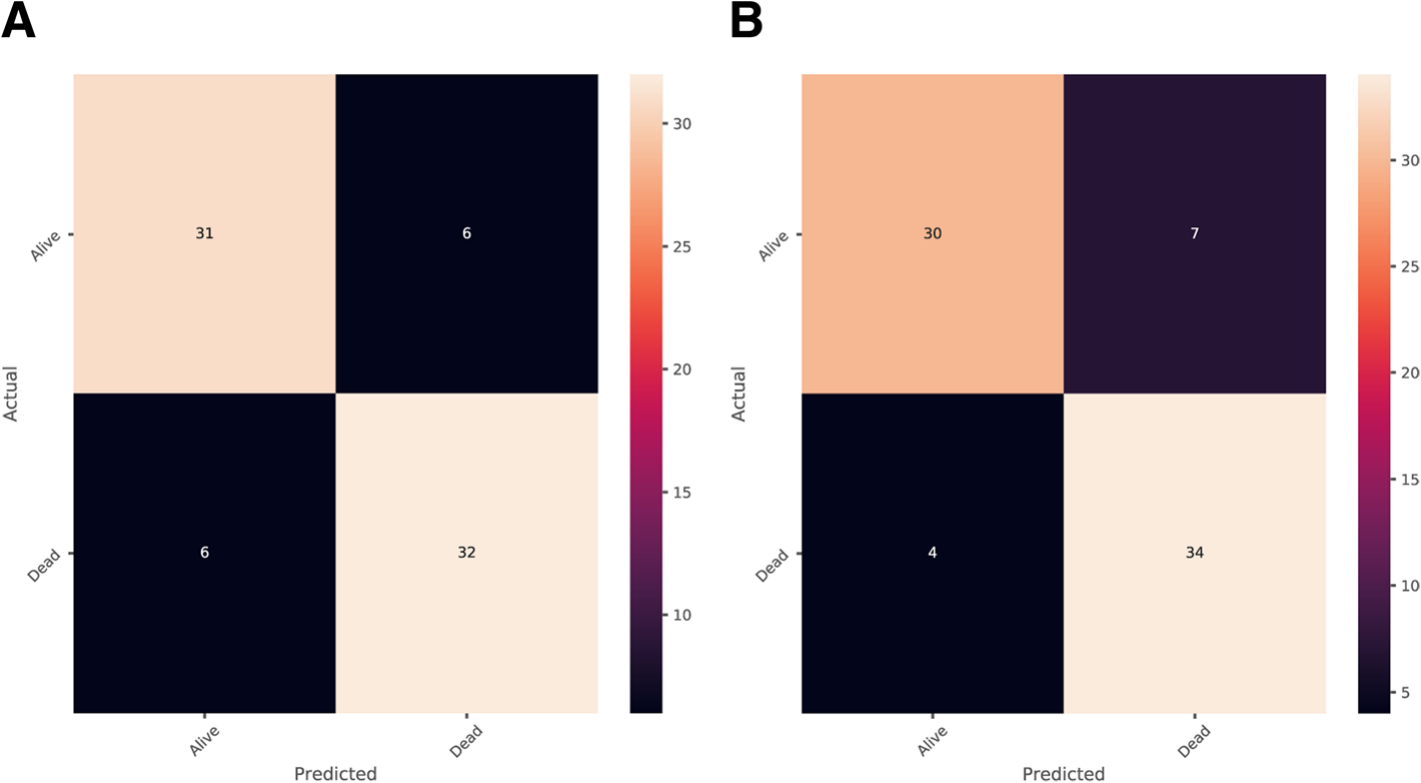
Confusion Matrix of Parameter Tuning Based on Different Evaluation. **a** Based on precision. **b** Based on recall. The horizontal line means the number of predicted in each group; the vertical line means the actual number of each survival group. The leading diagonal represents correct prediction; the minor diagonal represents incorrect prediction

**Fig. 3:**
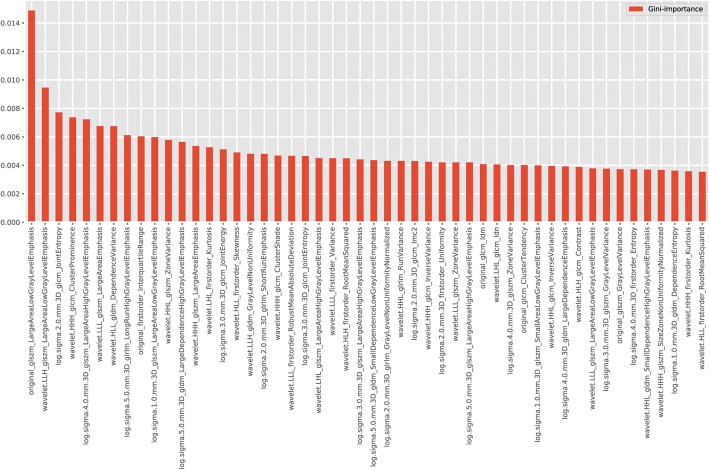
Fifty Features with Top Gini Importance Values. X axis is the name of features and Y axis represents the Gini-importance score

The construction of the classifier was conducted using a training set consisting of 223 patients with different survive status of NSCLC. Random forest and automatic parameters tuning were applied on the training data to obtain the optimal model. The partial results can be seen in Tables 2 and 3. The confusion matrixes of parameter tuning based on different evaluation standards could be seen in Fig. 2. Comparing the performance of these two models on the validation data, it could draw a conclusion that the model obtained based on recall standard outperformed slightly better than that of precision (The number of correct prediction was slightly larger and false negative was less). The final random forest classifier was constructed using the parameters: ‘max_depth’: 5, ‘max_features’: 20, ‘min_samples_split’: 3 and ‘n_estimators’: 100. Figure 3 displayed the fifty most important features generated in the random forest by Gini importance. As can be seen from the plot, two large area low gray level emphasis features ranked first and second respectively as the most important features in the prediction model but this did not mean that other features were much less important. Since when there were multiple correlated features, once a feature was selected the extent to which other features could lead to impurity decreasing dramatically.

**Table 2:** Model ranking based on mean precision score

Model	Mean Precision	Mean Recall	Mean Accuracy	Max Depth	Max Features	Min Samples Split	N Estimators
149	0.886	0.892	0.883	15	3	3	1100
252	0.882	0.91	0.888	25	10	3	100
164	0.878	0.901	0.883	15	5	3	500
154	0.878	0.892	0.879	15	3	5	900
233	0.878	0.847	0.861	25	3	10	1100

This table displayed the results of automatic hyper-parameters tuning based on two evaluation standards and ranked the models based on mean precision score. The last four columns represent the values of hyper-parameters of models

**Table 3:** Model Ranking Based on Mean Recall Score

Model	Mean Precision	Mean Recall	Mean Accuracy	Max Depth	Max Features	Min Samples Split	N Estimators
221	0.886	0.892	0.883	25	3	3	1100
279	0.879	0.892	0.874	25	20	5	700
153	0.884	0.883	0.879	15	3	5	700
225	0.884	0.883	0.879	25	3	5	700
146	0.878	0.883	0.874	15	3	3	500

This table displayed the results of automatic hyper-parameters tuning based on two evaluation standards and ranked the models based on mean recall scores. The last four columns represent the values of hyper-parameters of models

**Fig. 4:**
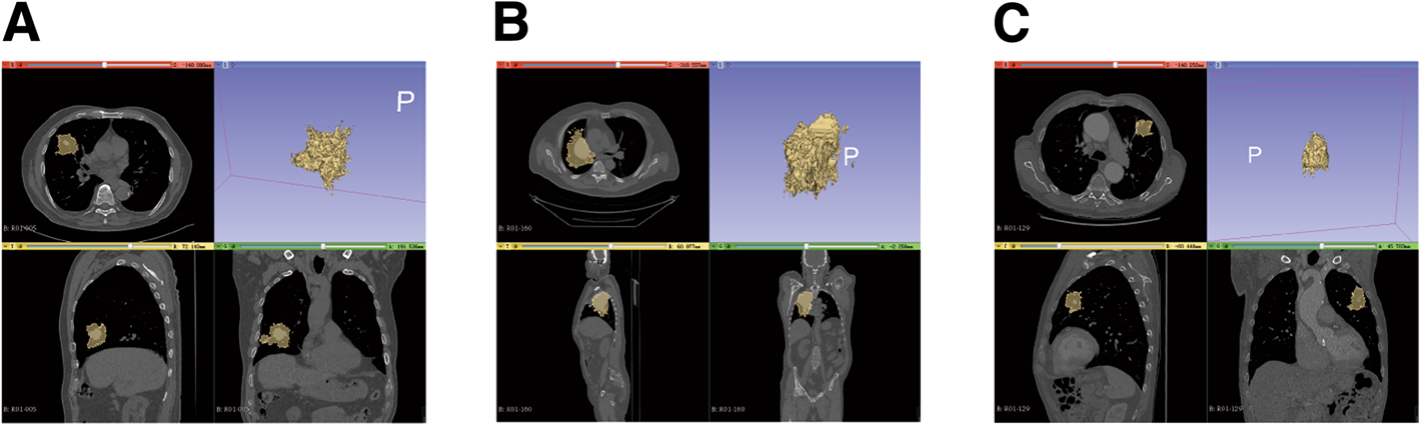
The Radiological Images of Three Certain Samples. **a-c** The patients’ living statuses from (**a-c**) are Alive, Dead, Dead

Figure 4 listed radiology images from separate body sections of three samples which were chosen randomly and all had certain information and features, such as histology, survival status, first order feature, GLSZM feature and etc. The survival status of (A) was alive, while that of (B) and (C) was dead. In common clinical diagnosis, researchers can make predictions based on the morphology of the lesions. As we can see through picture (A) and (B), size of tumor of (B) was significantly larger than that of (a), and clinicians with few experience could easily make difference of similar cases. However, once there was no distinguishing feature in the radiology images, clinicians hardly could diagnose the illness by naked eye. Through a set of comparison, this study found that a small part of image features had greater ability in prognosis. As a result, features extracted from radiology images were needed for further prognosis. Take (a) and (c) as an example, they are very similar in shape and size, whilst as shown in Table 4, difference in some of their features, like original_glszm_Large Area Low Gray Level Emphasis, wavelet.LLH_glszm_Large Area Low Gray Level Emphasis, wavelet. HHH_glcm_Cluster Prominence, wavelet.LLL_glszm_Large Area Emphasis and log.sigma. 1.0.mm.3D_glszm_Large AreLowGrayLevelEmphasis is remarkable.

**Table 4:** Basic information and the value of certain features of three cases

Features	R01–005	R01–006	R01–129
Case ID			
Histology	Adenocarcinoma	Adenocarcinoma	Adenocarcinoma
Survival Status	Alive	Alive	Dead
original_glszm_LargeAreaLowGrayLevelEmphasis	0.023323	0.330361	100.1903
wavelet.LLH_glszm_LargeAreaLowGrayLevelEmphasis	0.001637	0.008254	2.941066
wavelet.HHH_glcm_ClusterProminence	400.5173	413.4463	8.821475
wavelet.LLL_glszm_LargeAreaEmphasis	2.594846	8.408592	191.8787
wavelet.HLL_gldm_DependenceVariance	0.137999	1.28458	16.99916

It is shown in the table that different survival status corresponds to different level of feature, and it is noteworthy that the difference between them is distinguishing

### Decision threshold adjustment

**Fig. 5:**
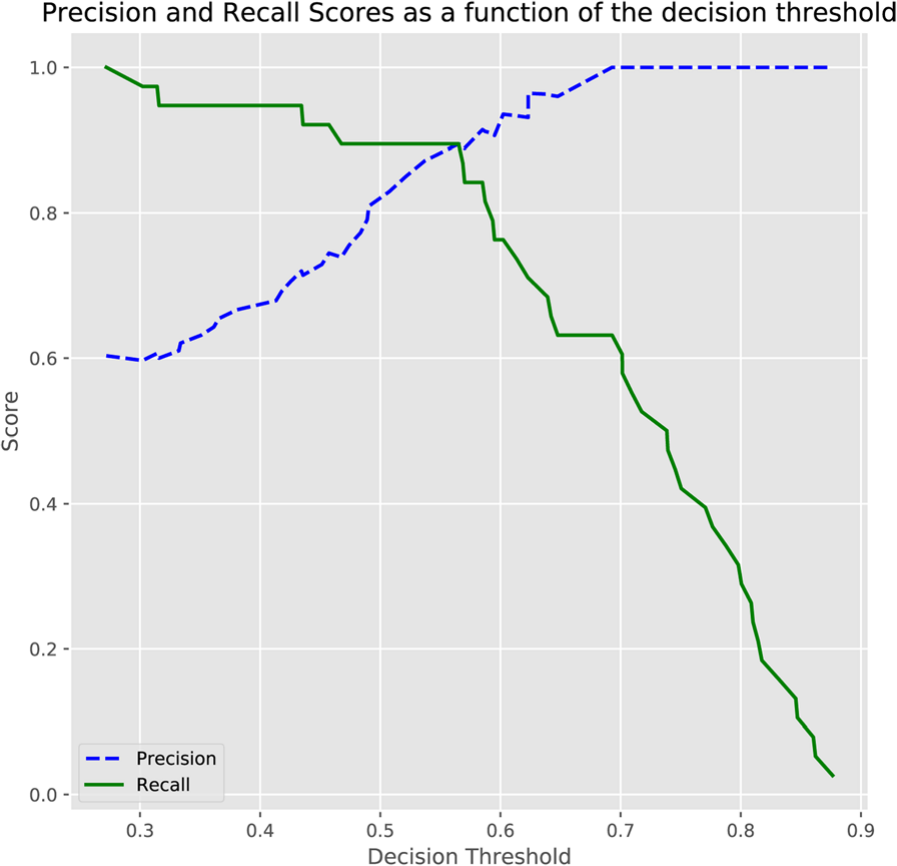
Precision and Recall Score as a Function of Decision Values. Blue dashed line: precision score; Green line: recall score. Y axis in the score value and X axis is decision threshold value. The intersection of the two curves are the optimal point where the trade-off of precision and recall is achieved

**Fig. 6:**
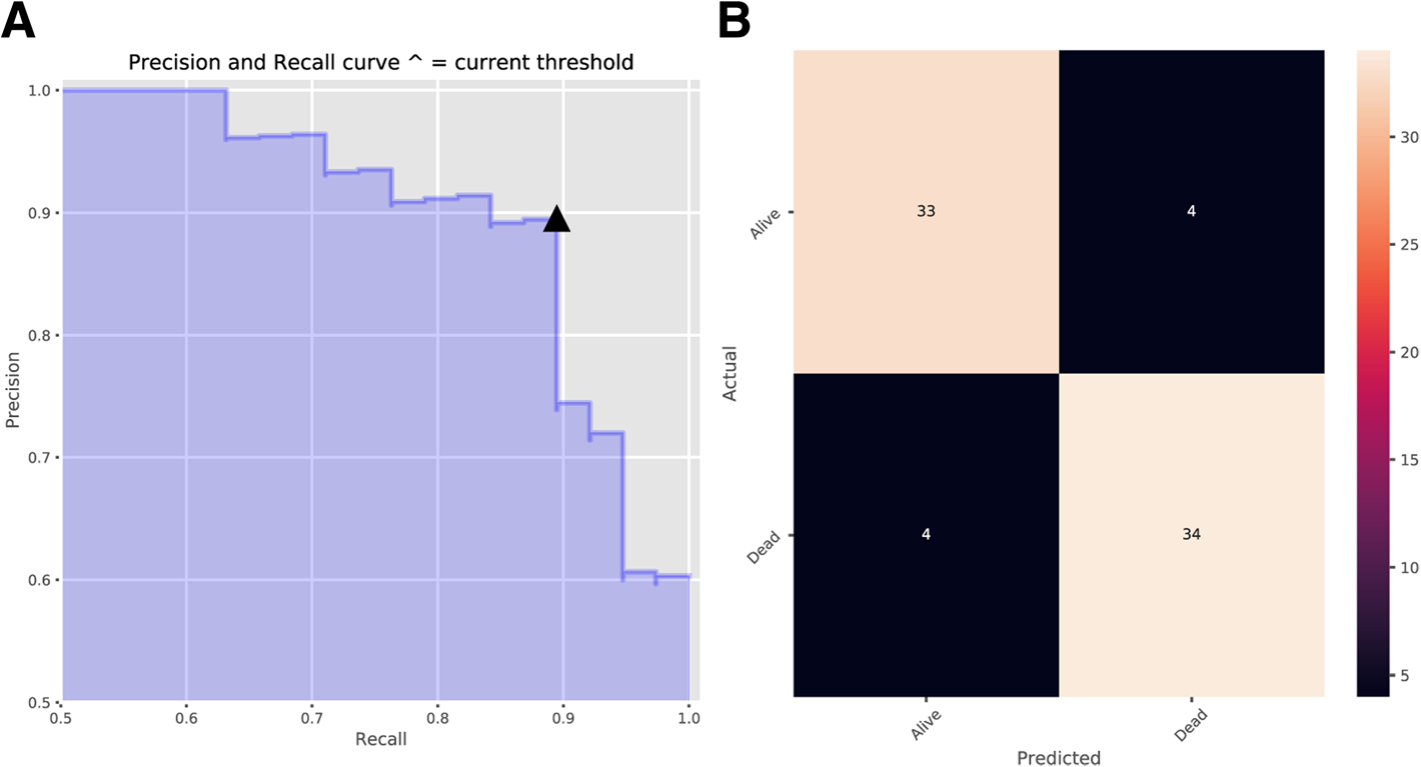
Precision and Recall with the Determined Decision Threshold. **a** Precision and Recall Curve (This curve shows how recall and precision changes as the decision threshold value changes. The triangle represents the decision threshold we chose). **b** Confusion Matrix. The horizontal line means the number of predicted in each group; the vertical line means the actual number of each survival group. The leading diagonal represents correct prediction; the minor diagonal represents incorrect prediction

After deciding the random forest classifier, we searched for the decision threshold for a trade-off between precision and recall. The default decision threshold in random forest was 0.5. Figure 5 showed precision and recall as a function of decision values, where x represented threshold value and y was the score of precision or recall. The optimal decision threshold was obtained as 0.56 from the intersection point and the precision of the model achieved nearly 90% when recall was around 90%, which was further verified by the precision and recall curve as well as the confusion matrix in Fig. 6.

### Performance of Radiomics prediction model

**Fig. 7:**
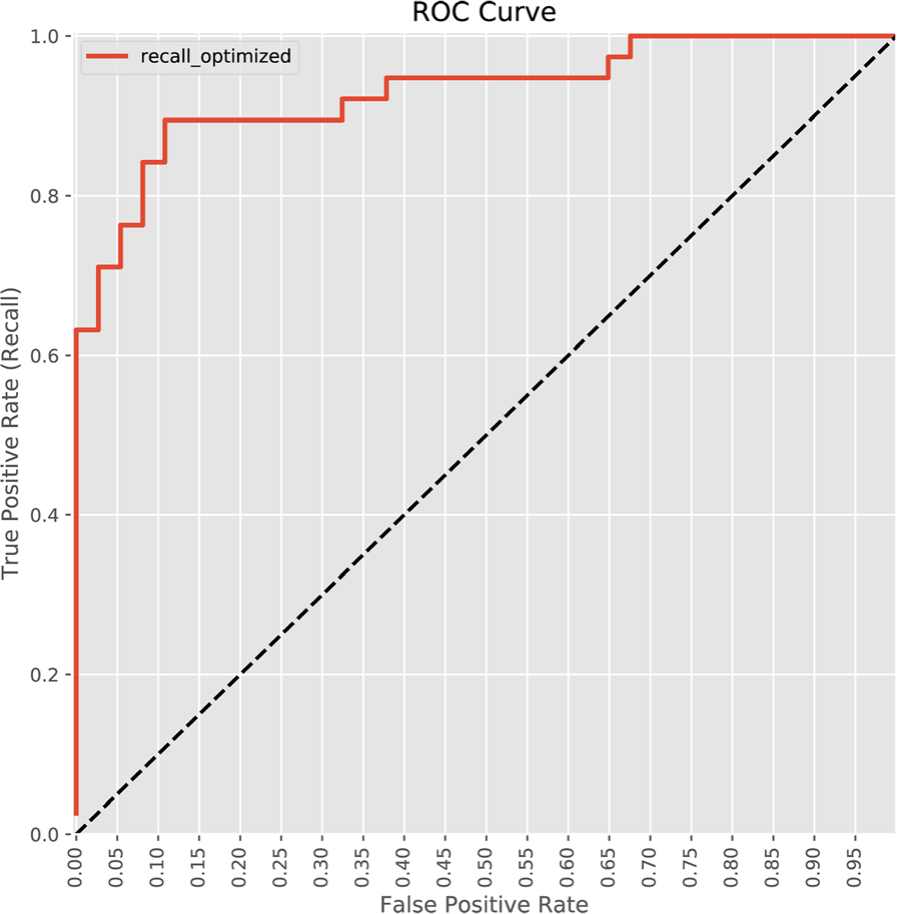
The ROC curve for Random Forest Model Performance on the Validation Data. X axis represents false positive rate ($\frac{\mathrm{FP}}{\mathrm{TN}+\mathrm{FP}}$) and Y axis is true positive rate ($\frac{\mathrm{TP}}{\mathrm{TP}+\mathrm{FN}}$). The diagonal dashed line means random prediction

The performance of the classifier constructed was validated according to the receiver operating characteristic (ROC) metrics in the validation set consisting 42 patients. Figure 7 presented the performance results (AUC: area under the ROC curve) obtained in the validation set for the radiomics model. The prediction accuracy was 89.33% (The percentage of correct classification). The AUC score for this model was 0.9296, which meant the model had a great ability of predicting being alive or being dead.

## Discussion

Radiomics has gained great attention as a potential method to promote personal medicine. Its image signatures derived from digital images are promising to help diagnostics and prognostics [24]. It has been shown that features such as texture, shape and intensity had prognostic power in independent data of lung and head-and-neck cancer patients since they were able to capture the intratumor heterogeneity [25]. Applying machine learning techniques on the output data from radiomics has also become a hot topic in oncology, personalized medicine and computer aided diagnosis since its compatibility with the big data generated from digital images [26].

This study intended to predict the non-small cell lung cancer survival status with radiomics features. A total of 1218 features were obtained after feature derivation using Pyradiomics. And those features captured the information about tumor shape, intensity and texture. Data imbalance is always a common problem in classification problem since most interested events like disease, network intrusion and etc. are rare. When sample size is large enough, slight or medium imbalance is not a big problem for training since there is enough information for learning from the minority class. However, when sample size is small, especially for decision tree the leaves that predict the minority class are likely to be pruned [27]. Thus, in this study, a SMOTE oversampling method was necessary to decrease the training fit error. The model was trained with automatic hyperparameters tuning aiming to make use of the best potential of our model. One common problem with hyperparameter tuning is overfitting, which means that the model performs well on the training data but poorly on the test data. This issue can be amended by using cross-validation where the model performance is evaluated by averaging k models. This study skipped the part of feature selection for two reasons: one was that the sample feature ratio was not low to introduce overfitting and another one was that random forest with parameters tuning was powerful since it optimized the number of tress and selected the best feature at each node. The final result of our study also proved that the model without feature selection had a great generalization on the test data.

Moreover, we studied how the adjustment of decision values impacted the precision and accuracy because a good classification model was not only evaluated on the accuracy. Precision and recall are further standards for model evaluation but there is a trade-off between them. Precision decreases as recall increases [28]. For survival status prediction, it is important to differentiate the death to stratify the patient into high risk group automatically. Thus, it might be more cost expensive to misidentify the high-risk patients. Without further test data validation, the result after decision threshold adjustment could be optimistic. However, this study gave a reference for the decision threshold of a non-small cell patient not being alive based on the radiomic feature. This may indicate that radiomics is promising into automatic computer aided patient risk stratification in a non-invasive way. For hyperparameters tuning, this study used grid search, which performed well in low dimensional space. When the dimension space is large or unknown, random search could be considered [2].

Despite of the satisfactory model performance, the study had a few limitations. First, since all training and testing data were acquired from one study, it may not be generalized to all cases [29] with a more heterogeneous dataset, and the accuracy might be lower than the current study. The extents to which the data we used are representative to the real situation also affect how well the trained model could perform in the practical use. Thus, different CT images from different sources are needed to construct a more rigorous and general classification model. The second limitation of the study is disproportion of patients at different disease stages. According to Table 2, the number of people with specific characteristic was far more than the rest, which means patient with a certain stage of the cancer might have more images or more pathologic images. For instance, there were 154 Adenocarcinoma patients, 29 Squamous cell carcinoma patients, and 3 not otherwise specified (NOS) patients.

For future research, more data from diverse patients’ background, different databases, and multiple image modalities should be utilized for further testing and validation. Other mathematical model can be developed to improve feature extraction. Our model can be adopted to improve the performance of classifier. The most relevant features can provide useful information for future exploration to develop a better detection method. Also, with larger dataset, different classification criteria can be tuned according to the different types of lung cancer and disease stages.

## Conclusion

To conclude, this study intended to construct a survival status classifier with automatic hyperparameters tuning. In order to optimize classification outcomes, the tuning of decision threshold can serve as a reference for future work. Our classification methods has the potential to contribute to a survival prediction model, which is beneficial to better palliative care and treatment decision.

## Additional file

Additional file 1: **Table S1.** Demographic Information of the patients. (DOCX 15 kb)

## Abbreviations

AUC: Area under the curve
GLRLM: Gray Level Run Length Matrix
GLSZM: Gray Level Size Zone Matrix
NSCLC: Non-small cell lung cancer
ROC: Receiver operating characteristic
SMOTE: Synthetic minority over-sampling
TCIA: The cancer imaging archive
