# Supplementary material for: A biomarker basing on radiomics for the prediction of overall survival in non–small cell lung cancer patients
Source: Respir Res. 2018 Oct 10;19:199. doi: 10.1186/s12931-018-0887-8 (PMC6180390; doi:10.1186/s12931-018-0887-8)
Supplement: Supplementary file 1 — Table S1. Demographic Information of the patients. (DOCX 15 kb) [file 12931_2018_887_MOESM1_ESM.docx]

**Supplementary Table 1. Demographic Information of the patients**

| **Parameters** |  | **Total** | **Dead** | **Alive** |
| --- | --- | --- | --- | --- |
| **Gender** | male | 106 | 28 | 78 |
|  | female | 38 | 5 | 33 |
| **Age** | <60 | 20 | 4 | 16 |
|  | ≥60,<70 | 57 | 10 | 47 |
|  | ≥70,<80 | 49 | 15 | 34 |
|  | ≥80 | 18 | 4 | 14 |
| **Smoking Status** | Nonsmoker | 21 | 4 | 17 |
|  | Former smoker | 93 | 21 | 72 |
|  | Current smoker | 30 | 8 | 22 |
| **Histology** | Adenocarcinoma | 112 | 26 | 86 |
|  | Squamous cell carcinoma | 29 | 6 | 23 |
|  | NSCLC NOS | 3 | 1 | 2 |
| **T stage** | Tis | 2 | 0 | 2 |
|  | T1 | 66 | 15 | 51 |
|  | T2 | 50 | 13 | 37 |
|  | T3 | 20 | 5 | 15 |
|  | T4 | 6 | 1 | 5 |
| **N stage** | N0 | 115 | 19 | 96 |
|  | N1 | 12 | 6 | 6 |
|  | N2 | 16 | 8 | 8 |
| **M stage** | M0 | 144 | 34 | 110 |
|  | M1 | 0 | 0 | 0 |

NOS=Not Otherwise Specified

This table displayed the detailed clinical information.
